# Cognitive Strategies and Physical Activity in Older Adults: A Discriminant Analysis

**DOI:** 10.1155/2018/8917535

**Published:** 2018-04-05

**Authors:** Nathalie André, Claude Ferrand, Cédric Albinet, Michel Audiffren

**Affiliations:** ^1^Centre de Recherches sur la Cognition et l'Apprentissage, UMR CNRS 7295, Université de Poitiers, Poitiers, France; ^2^Maison des Sciences de l'Homme et de la Société, USR CNRS 3565, Université de Poitiers, Poitiers, France; ^3^EA 2114, Psychologie des âges de la vie, Université François Rabelais, Tours, France; ^4^Laboratoire Sciences de la Cognition, Technologie, Ergonomie (SCoTE), Université de Toulouse, INU Champollion, Albi, France

## Abstract

**Background:**

Although a number of studies have examined sociodemographic, psychosocial, and environmental determinants of the level of physical activity (PA) for older people, little attention has been paid to the predictive power of cognitive strategies for independently living older adults. However, cognitive strategies have recently been considered to be critical in the management of day-to-day living.

**Methods:**

Data were collected from 243 men and women aged 55 years and older living in France using face-to-face interviews between 2011 and 2013.

**Results:**

A stepwise discriminant analysis selected five predictor variables (age, perceived health status, barriers' self-efficacy, internal memory, and attentional control strategies) of the level of PA. The function showed that the rate of correct prediction was 73% for the level of PA. The calculated discriminant function based on the five predictor variables is useful for detecting individuals at high risk of lapses once engaged in regular PA.

**Conclusions:**

This study highlighted the need to consider cognitive functions as a determinant of the level of PA and, more specifically, those cognitive functions related to executive functions (internal memory and attentional control), to facilitate the maintenance of regular PA. These results are discussed in relation to successful aging.

## 1. Introduction

Generally, physical activity adherence is examined by social psychologists and health psychologists to prevent sedentariness. Reviews of the gerontological literature on this topic reveal a number of socioeconomic, demographic, psychological, attitudinal, and accessibility correlates and/or determinants of physical activity (PA) [1–5]. For instance, when focusing on independently living elderly people, van Stralen et al. [2] and Koeneman et al. [3] reported several moderate to strong determinants of regular PA such as age, gender, education, perceived health and depression, baseline PA behavior, barriers' self-efficacy, benefits of regular PA, and social support. However, adherence also requires continuous effort and strategies underpinned by executive functions to maintain the behaviors involved in the healthy management of day-to-day living such as PA [6], diet regimen, and medication [7].

It is now well known that cognitive functions undergo a decline during aging [8], and this decline is often associated with the use of compensatory cognitive strategies that could help aging people cope with their diminished cognitive performances. For instance, some elderly people use external memory strategies, such as writing a shopping list on a piece of paper to compensate for a decline in episodic memory. In some cases, these strategies can be considered counterproductive in a long-term perspective because external memory aids could stimulate negative stereotype related to aging by reducing perceived efficacy and perceived control on memory [9]. These strategies could lead the individual to reduce the cognitive resources used to initiate behaviors and, consequently, not to stimulate or maintain their self-regulation ability. Thus, each time an elderly person chooses to write his/her shopping list on a piece of paper to avoid forgetting an item during shopping, he/she does not stimulate his/her encoding and retrieval memory systems and consequently undermines his/her episodic memory in the long term. Fortunately, not all compensatory cognitive strategies reduce the cognitive resources invested to cope with a problem but rather use alternate or vicarious cognitive systems, for instance, internal memory strategies [10]. In another respect, Lachman and Andreoletti [11] reported that when metacognitive beliefs were lacking, older adults were less strategic in solving problems, and this lack of mastery experiences reduced self-efficacy and resulted in less autonomy and more dependency.

The relationship between cognitive functioning and adherence has been studied either by examining populations with cognitive impairments [12, 13] or by training populations in cognitive processes [14–18]. These studies have been principally carried out in the context of medication adherence. For instance, studies have reported that older people with cognitive dysfunctions show poor medication adherence compared to healthy older people [12] and that working memory ability or habitual prospective memory facilitated recall of medication instructions [19, 20]. In their systematic review on the efficacy of interventions to increase medication adherence among community-dwelling seniors with cognitive impairments, Kröger et al. [13] have shown that reminder strategies are promising to improve adherence. Finally, Karr et al. [21] have shown that regardless of the population and executive functions targeted, cognitive training (specific or through physical activity) has positive effects on adherence. To our knowledge, very few studies have investigated the links between cognitive functioning and adherence to physical activity with healthy older adults [22–24]. As highlighted by McDonald-Miszczak et al. [25], despite a lack of clear identification of the cognitive functions implied in the adherence process, the research literature has identified several cognitive processes that can be considered to be responsible for psychological disturbances and nonadherence when they are impaired. Recently, Olson et al. [26] reported that cognitive functions such as working memory, controlled inhibition, attention, and task switching predicted PA adherence 6 months after the end of a PA program in older adults with metabolic diseases. Other studies have identified prospective memory [14, 27], metamemory [8, 28], and attentional control [22, 29, 30] as determinants of high levels of cognitive functioning. It can be reasonably hypothesized that remembering planned sessions during the week (prospective memory) and inhibiting the desire to stop exercise when it is difficult (controlled inhibition) are two high-level cognitive functions required to maintain the regular practice of a moderate to vigorous level of PA over weeks, months, or years. On one hand, prospective memory is related to long-term memory and declarative memory, and it describes the ability to plan and successfully execute delayed intentions in the future [31]. People in general, and more particularly aging people, use strategies to facilitate and/or solve a prospective memory task. Studies on prospective memory have demonstrated that this cognitive process facilitates medication adherence [27]. Metamemory can be defined as the control and regulation of strategies necessary to address such memory tasks [32]. More precisely, metamemory refers to two categories of strategies: (1) internal strategies, such as using mental imagery, recalling contextual cues, associating cues, or listing events; and (2) external strategies, such as making shopping lists or writing down appointments on a calendar. Gould et al. [27] have reported that older adults preferentially use internal memory strategies—although this relationship is mediated by depression and concerns about memory efficacy—to remember their medication but external memory strategies for everyday situations. On the other hand, attentional control can be conceptualized as the ability to filter information by focusing on pertinent cues and inhibiting nonpertinent cues [33]. Controlled inhibition can be considered a component of attentional control and concerns the ability to repress actions, thoughts, emotions, or impulses. Attentional control was greatly examined by Hillman et al. [22, 23] in relation to physical activity, and the authors reported that PA activates the attentional system and mobilizes attentional resources.

Many epidemiological studies have examined the benefits of PA on cognition and have shown a positive relationship between higher levels of PA and a reduced risk of cognitive impairment (for a review, see [34, 35]). For instance, some studies based on large sample sizes and long follow-up periods have shown that older adults who had previously engaged in higher levels of PA were likely to perform better on cognitive tasks when compared with participants with previously lower PA levels [36, 37]. Consequently, PA is hypothesized to have a protective effect on cognitive decline in older adults. The brain structures most negatively affected by aging are also those that benefit the most from physical activity (e.g., Colcombe et al. [38]). For instance, the prefrontal cortex and the hippocampus, which underlie executive functions and encoding of information in episodic memory, respectively, are more severely affected by aging than brain structures involved in procedural memory [39]. Because of this positive effect of PA on cognitive functions, it can be expected that active older adults will show better cognitive functioning and use better cognitive strategies than inactive ones. Reciprocally, better functioning in executive functions may facilitate the maintenance of new healthy behaviors such as a physically active lifestyle [40], and consequently, it could be expected that aging people who use more effective cognitive strategies maintain a higher level of regular physical activity.

The purpose of this study was to identify predictors of the level of PA among a sample of independent-living older healthy people from the PRAUSE project. We are particularly interested in the perception of use of cognitive strategies rather than the effectiveness of these strategies. It can be expected that because PA reduces the decline of cognitive functions and because the efficient functioning of cognitive functions facilitates the use of functional strategies, we should observe differences between active and inactive older adults in the use of cognitive strategies. As suggested by West et al. [41], cognitive functions meaningfully influence thought and actions and determine adaptive coping to challenges in everyday life. The main objective of the present study was to examine whether cognitive strategies related to executive functions and prospective memory would predict the level of engagement in physical activity in older persons.

## 2. Methods

### 2.1. Description of Sample

The present study is a part of a French regional survey, a multidisciplinary research project entitled Seniors' Autonomy Preservation in Poitou-Charentes (PRAUSE). The inclusion criteria to be eligible for the recruitment into PRAUSE were the following: (1) living in Poitou-Charentes; (2) being aged 55 years or over; and (3) not being institutionalized and not under guardianship or trusteeship. Participants were invited to take part in the study by mail and phone. Project feasibility was tested with a pilot study, and three waves of data collection were necessary to include a total of 466 participants. Each participant included in the study performed one to three sessions of data collection according to his/her motivation. For the purposes of this study, only 243 participants out of 466 completed all the data required to test our hypotheses. This sample of participants included 41.57% males and had a mean age of 74.02 (SD = 9.61) years.

### 2.2. Compliance with Ethical Standards

All participants signed an informed consent form. The three data collection waves occurred between 2011 and 2013. All participants were visited at home, and all face-to-face questionnaires were administered by investigators who received individual training for all data collection. The protocol of PRAUSE was approved by two national ethics committees: (1) the “general interest and statistical quality” label from the French National Council of Statistical Information (CNIS, visa no. 2012X907RG) and (2) the French National Commission on Informatics and Liberty (authorization no. 1593815).

### 2.3. Demographic and Health Variables

Questionnaires were completed with data including age, gender, perceived health status, education, depression, decisional balance, and BMI. Perceived health status was evaluated with a visual analogue scale from EuroQol-5D and consisted of assessing his/her perception of health from “best known health status” to “worse known health status.” The *Decisional Balance* questionnaire was based on Marcus' version and adapted to physical activity. It consisted of two constructs that underlie cognitive and motivational aspects of human decision making. These constructs have been labeled the pros and cons of exercising, and they were each measured with 8 items. Participants responded to each item on a 5-point Likert-type scale ranging from totally agree (1) to totally disagree (5). Based on Bandura's guidelines [42] and the identification of main barriers in old age reported in the literature [43, 44], six barriers' self-efficacy were included based on health aspects (pain and fatigue), motivational determinants (too busy and nobody to practice with), and environmental factors (weather and accessibility). Participants rated answers on a 5-point scale ranging from 1 (not confident to overcome) to 5 (extremely confident to overcome). Cronbach's alpha values were .76 for pros, .83 for cons, and .68 for barriers' self-efficacy. Depression GDS, Depressive symptoms were assessed using the 30-item Geriatric Depression Scale validated in French by Bourque et al. [45]. A score of 0–9 is normal, a score of 10–19 indicates slight depression, and a score of 20–30 corresponds to severe depression.

### 2.4. Physical Activity Measure

 The level of current PA was evaluated with the Historical Leisure Activity Questionnaire (HLAQ) [46]. This questionnaire was used to assess the history of PA weighted by relative intensity, and as suggested by Kriska et al. [46], the list of activities was adapted to the population. The HLAQ was previously used in French studies that demonstrated relationships between executive functions and level of physical activity [47, 48]. Participants were asked to report the frequency, type, intensity, and hours of PA performed during the present year. Using the Compendium of Physical Activities Tracking Guide 2011 [49], we obtained a specific metabolic equivalent (MET) for each PA. According to the HLAQ data and the compendium, we calculated the average energy expenditure (METs-h/week) for each participant. According to the WHO recommendations, we classified the participants above 7.5 METs-h/week in the active group and those below 7.5 METs-h/week in the inactive group.

### 2.5. Cognitive Variables

Cognitive strategies were measured using a questionnaire adapted from three scales: (1) the Metamemory in Adulthood scale [50], (2) the Thought Control Questionnaire [51], and (3) the Attentional Control Scale [52] (Table 1). Metamemory concerns knowledge of memory functioning, beliefs and affects about memory, as well as monitoring and autoregulation during memory activity [50]. The metamemory in adulthood scale contains eight subscales, from which two were selected and adapted for the study: the external strategy subscales (e.g., memos and calendar) and the internal strategy subscales (e.g., mental imagery and word associations). These scales evaluate the means used by people to more easily find information stored in prospective and episodic memory. The external strategy subscales include 6 items and the internal subscale 9 items. Cronbach's alphas for these two cognitive strategies were 0.66 and 0.70, respectively. The thought control questionnaire contains five subscales designed to assess people's tendency to use a variety of thought control strategies in everyday life. Two subscales were selected and adapted for the needs of the study: the reappraisal subscale (e.g., I try to reinterpret the thought) and the distraction subscale (e.g., I occupy myself with work instead). The reappraisal subscale includes 5 items and the distraction subscale 6 items. Cronbach's alphas for these two cognitive strategies were 0.83 and 0.75, respectively. The short-form Attentional Control Scale consisted of a 12-item self-report [52] measure combining attentional focusing that requires voluntary control over behaviors and attentional shifting that is related to performance on switching tasks. Only the attentional focusing strategy was evaluated in the study because of the link we posited with PA. This scale includes 6 items. Participants rated answers on a 5-point scale ranging from 1 (never used) to 5 (always used) for the five strategy subscales. Cronbach's alpha for this scale was 0.66.

### 2.6. Data Analysis

Descriptive statistics were examined to characterize the study population. Correlation analyses and *t*-tests were used to obtain additional descriptive information and to identify a preliminary set of predictor variables to be included in the discriminant analysis. Variables with a significant difference of *p* < 0.05 as determined by the above tests were entered into stepwise discriminant analysis. Then, a stepwise discriminant analysis was performed to determine if active and inactive participants could be discriminated based on the following variables: age, gender, body mass index (BMI), perceived health status, depression, barriers' self-efficacy, and cognitive strategies (internal memory and attentional control).

## 3. Results

Table 2 shows distributions of sociodemographic characteristics and other covariates. Participants who were active had a mean age of 71.45 (SD = 8.65), had a mean BMI of 26.88 (SD = 4.94), perceived themselves on average in better health (75.46, SD = 14.27), perceived themselves on average as capable of overcoming barriers (38.51, SD = 9.83), were not depressed (6.48, SD = 4.80), used on average more internal memory strategies (30.28, SD = 5.21), and were not easily distracted (19.41, SD = 4.37). Participants who were inactive had a mean age of 77.66 (SD = 9.75), had a mean BMI of 28.70 (SD = 4.70), perceived themselves in worse health (66.37, SD = 18.01), perceived themselves as less capable of overcoming barriers (31.85, SD = 10.06), used on average less internal memory (28.76, SD = 6.61), and had difficulties in concentrating (20.46, SD = 5.04).

The emphasis of this analysis was on understanding how these variables were related to each other to determine the level of physical activity. We used discriminant analysis to determine the linear combination of predictor variables that best classified the cases into the two groups. The stepwise discriminant analysis (Table 3) showed that Wilks' lambda, as a test of discriminant function, was significant (lambda = 0.736; *χ*^2^ = 72.051, df = 8, *p* < 0.001) and selected the five following variables as determinants of physical inactivity (based on structure matrix loading): older (−0.626), perceived poor health (0.442), less use of internal memory strategies (0.213), attentional control (−0.196), and poor confidence to overcome barriers to PA practice (0.555). Wilks' lambda, which describes the proportion of total variance in the discriminant score not explained by differences between groups, was significant, indicating that it is unlikely that participants who were inactive and those who were active had the same means on the discriminant functions generated from the prediction equation.

Table 4 summarizes the group membership results of the classification routine. Of the 104 participants who were inactive, 63 (60.6%) were correctly classified as inactive, and of the 139 participants who were active, 113 (81.3%) were correctly classified as active based on the selected variables. The overall percentage of the level of PA classifications was 73%, reflecting a 23% improvement over chance alone. Of the variables investigated, age was the most discriminating and barriers' self-efficacy the least.

## 4. Discussion

Sedentariness and inactivity are legitimate problems in older adults because these behaviors lead to several health problems such as more pronounced cognitive decline, sarcopenia, or social isolation. Most studies that have examined the factors associated with sedentariness or inactivity have focused on psychosocial predictors. However, cognitive functions have recently been considered in adherence to treatment medication [12, 13], but to our knowledge, no study has examined the predictive value of cognitive strategies in adherence to PA in healthy older adults. The aim of the present study was to identify cognitive and psychosocial determinants of the level of PA in independently living healthy older adults in France.

First, no difference was observed between active and inactive healthy older adults when considering level of education, knowledge concerning the benefits of regular PA, the use of external memory strategies, cognitive and behavioral distractions, or reappraisal. These results are partially consistent with previous literature reviews examining the relationships between sociodemographic and psychosocial determinants and the level of PA [2, 3, 53]. The results concerning knowledge about the benefits of regular PA suggest that information provided by the campaigns of prevention are well retained by individuals in general, regardless of their level of engagement in regular PA. As suggested by Gross and Rebok [53], however some demographic predictors such as gender, pros of exercising, and level of education could be used in high-risk populations, these predictors are not necessarily significant in healthy populations. It is interesting to note that, in the present study, active older adults scored lower on the pros scale than inactive ones. This result could indicate that inactive older adults overestimate pros but are not interested or concerned by these potential benefits. Three out of five cognitive strategies showed no significant differences between active and inactive older adults, with external strategies being more used than the other strategies by older adults in general (*M* = 4.82; close to “always used”), followed by distraction (*M* = 3.43; close to “sometimes used”) and reappraisal strategies (*M* = 2.36; close to “rarely used”), the latter being the least used and referring to problem solving. These results could be interpreted in two ways: (1) maintaining a high level of PA involves specific cognitive functions and strategies and (2) practicing a high level of PA facilitates the use of higher cognitive functions and strategies.

This study also revealed some interesting results concerning predictors of the level of PA that could contribute to reinforce interventions intended to help older people adhere to long-term PA practice. The discriminant analysis revealed that among the variables that emerged as significantly predictive of the level of PA, age, perceived health, barriers' self-efficacy, internal memory strategies, and attentional control strategies predominantly discriminated between active and inactive older participants in successfully classifying 73% of participants. Concerning sociodemographic and psychosocial variables, these results are congruent with previous studies. First, several studies [2, 3] moderately suggested that males are generally more active than females. Unlike these studies, gender was not a predictor of the level of PA in the present study. In a recent systematic review [54], it appears that gender differences in walking is attenuated for old adults (>70 years). Second, perceived health status was presently identified as a determinant of engagement in PA but not depression. However, McHugh and Lawlor [55] have reported an overadditive interaction of these two variables on hours of exercise per week such that high perceived health status combined with low levels of depression resulted in the highest levels of exercise. In the present study, even if depression was correlated to the level of PA, this variable did not discriminate between active and inactive older adults. Consequently, it could be suggested that (1) perceived health status and depression share a common variance, certainly because global perceived health status includes a component of mental health status such as depression (*r* = −0.365 for this study) or (2) perceived health status is a better predictor of PA than depression. Third, and finally, barriers' self-efficacy, which appeared as a predictor of the level of PA in the present study, were already reported as predictive of adherence to PA [56], more precisely, in elderly women in a 6-month strength training program. In this last study, barriers' self-efficacy were a good predictor of the first three months of exercise adherence and remained a significant predictor after six months. In the present study, barriers' self-efficacy were a good predictor of regular and habitual physical activity. Both studies suggest that barriers' self-efficacy are a good predictor of exercise adherence from a long-term perspective, possibly because this variable positively influences motivation to practice by increasing effort and persistence as well as enhancing attention paid to tasks.

The important contribution of this study is the discriminant power of cognitive strategies, indicating that active older people perceive themselves as using more internal memory strategies—assessing prospective and episodic memory—and as more able of using controlled inhibition than inactive ones. In other words, these perceptions would favor engagement in physical activity because they would reinforce positive perception of one's own efficacy to interact with environment. Another explanation could be that practicing moderate to high level of physical activity improves episodic memory and consequently facilitates the use of internal memory by older adults and their perception of using these strategies. Because this study was cross-sectional, we cannot define a causal relationship between these variables. Notwithstanding, a first assumption would be that older adults who have better memory abilities and/or better attentional control engage themselves more easily in active behaviors. This explanation is reinforced by previous research that has reported that the use of memory strategies facilitates the management of daily living activities [7], medication adherence [19, 26], and PA [15]. To our knowledge, only the study carried out by Olson et al. [26] reporteda relationship between attention and adherence to PA and postulated that controlled inhibition is critical to successful behavior changes. In other words, attentional control ability is important for switching between goal-directed plans and environment-based responses. According to the temporal self-regulation model proposed by Hall and Fong [6], executive functions are involved in PA behavior. However, these authors did not provide any details concerning the specific executive functions involved in adherence, such as planning, cognitive flexibility, or controlled inhibition. The present results clearly show that specific cognitive functions are associated with PA behavior: presumably, prospective memory related to internal memory strategies and controlled inhibition related to attentional control strategies. A second assumption would be that by being active, older adults increase their memory abilities and their attentional control. For instance, Winnecke et al. [30] reported that individuals with higher levels of PA showed better attentional control capabilities than those who are less active. This result confirms the proposition made by Hillman et al. [23] that PA can activate the attentional system and mobilize attentional resources. From that perspective, other studies showed that controlled inhibition seems to benefit more selectively from PA than from other executive functions [57, 58]. Studies on memory training programs reported that memory strategies are needed to maintain regular activity with a low investment of cognitive resources [59]. For instance, Wolff et al. [59] reported that action planning, defined by the authors as being strongly related to memory strategies, facilitates the enactment of health behavior in daily life. What is interesting to note here is that memory strategies are not necessarily associated with high prospective memory ability. In other words, physically active older adults might compensate for cognitive deficits by recruiting larger frontal areas relevant for memory and/or attentional control strategies. This compensatory mechanism would not necessarily consist of an increase of the efficiency of these functions but in a better use of the cognitive strategies. These strategies could, in turn, facilitate the day-to-day management of life and perceived health. There is now substantial literature demonstrating this reciprocal effect, and our results confirm and specify the theoretical position taken by Hall and Fong [6] on the mutual reinforcement of executive functions and PA over time. Our results argue in the direction of this model, and particular attention should be paid to the cognitive strategies involved according to the targeted behavior. The present study has certain limitations. First, the study employed cross-sectional data, precluding any assumptions concerning causality between the five variables selected as predictors and the level of PA. Moreover, those variables and the level of PA might have changed over time. Further analysis of longitudinal data is warranted to clarify the relationship between changes in the five predictors obtained and PA. Another limitation to this study is its reliance on self-reported data on the five selected variables and PA.

## 5. Conclusion

Rather than seeing older people as lacking motivation for physical activities, this study identified cognitive and psychosocial predictors of the level of PA to evaluate the likelihood of relapses or quitting and to reduce the risk factors through interventions among older people at increased risk of inactivity. Regardless of whether age is an irreversible process, this study raises awareness of the need to consider cognitive strategies as facilitators to engage in durable PA. The development of specific cognitive strategies related to cognitive functions seems a relevant element to facilitate the management of daily life and preservation from sedentariness.

## Figures and Tables

**Table 1:** Cognitive strategy questionnaires used in the study.

*External memory strategies*
I keep a list or otherwise note important dates, such as birthdays and anniversaries
I write shopping list to help me remember
I write appointments on a calendar to help me remember them
I routinely keep things (keys or glasses) in a familiar spot, so I will not forget them when I need to locate them
I post reminders of things I need to do in a prominent place, such as on bulletin boards or note boards
When I want to take something with me, I leave it in an obvious, prominent place, such as putting my suitcase in front of the door

*Internal memory strategies*

When I am looking for something I have recently misplaced, I try to retrace my steps in order to locate it
When I want to remember something, I concentrate hard on it
When I try to remember a telephone number, I mentally repeat it to myself
I think about the day's activities at the beginning of the day, so I can remember what I am supposed to do
I make mental images or pictures to help me remember an event or an individual
I try to relate something I want to remember to something else hoping that this will increase the likelihood of my remembering later
When I have trouble remembering something, I try to remember something similar in order to help me remember

*Reappraisal*

When I experience an unpleasant/unwanted thought, I challenge the thought's validity
When I experience an unpleasant/unwanted thought, I analyze the thought rationally
When I experience an unpleasant/unwanted thought, I try to reinterpret the thought
When I experience an unpleasant/unwanted thought, I try a different way of thinking about it
When I experience an unpleasant/unwanted thought, I question the reasons for having the thought

*Distraction*

When I experience an unpleasant/unwanted thought, I call to mind positive images instead
When I experience an unpleasant/unwanted thought, I occupy myself with work instead
When I experience an unpleasant/unwanted thought, I think pleasant thoughts instead
When I experience an unpleasant/unwanted thought, I do something that I enjoy
When I experience an unpleasant/unwanted thought, I think about something else
When I experience an unpleasant/unwanted thought, I keep myself busy

*Attentional control*

When concentrating on something and there are noises around me, I ask for silence
When I read, I look for a calm area where I will not be distracted by people around me
When I am working hard on something and I am distracted by events around me, I try to isolate myself
When trying to focus my attention on something and thoughts distracting me, I chase them out of my mind
When concentrating on something and hunger or thirst distracts me, I satisfy my need so that it does not worry me anymore
When concentrating on something, I turn off TV or radio

**Table 2:** Sociodemographic, health, motivational, and cognitive strategies for active versus inactive participants. The last column shows the correlations between the variable and the level of physical activity for the population sample examined in this study (*n*=243).

Variables	Active (*n*=139) mean (SD)	Inactive (*n*=104) mean (SD)	*p*	Correlation coefficient
✓^∗^Age (years)	71.45 (8.65)	77.66 (9.75)	Ϯ	−0.35Ϯ
✓^∗^Gender (M/F)	71/68	29/75	Ϯ	0.23Ϯ
✓Education	10.52 (3.66)	10.20 (3.80)	ns	0.05
✓^∗^BMI	26.88 (4.94)	28.70 (4.70)	Ϯ	−0.18Ϯ
✓^∗^Perceived health status	75.46 (14.27)	66.37 (18.01)	Ϯ	0.26Ϯ
✓^∗^Depression	6.48 (4.80)	9.067 (5.05)	Ϯ	−0.14Ϯ
*Decisional variables*				
✓Pros	15.84 (6.14)	17.27 (6.10)	ns	−0.11
✓^∗^Barriers' self-efficacy	38.51 (9.83)	31.85 (10.06)	Ϯ	0.36Ϯ
*Cognitive strategies*				
EMS (from 6 to 30)	23.86 (4.02)	24.30 (4.42)	ns	−0.06
^∗^IMS (from 9 to 45)	30.28 (5.16)	28.76 (6.61)	Ϯ	0.12
^∗^ACS (from 6 to 30)	19.43 (3.68)	20.46 (5.04)	Ϯ	−0.11
DS (from 6 to 30)	20.60 (4.94)	20.21 (5.40)	ns	0.04
RS (from 5 to 25)	14.58 (4.05)	13.62 (4.56)	ns	0.11

✓Variables reported as moderately to strongly significant in the literature. ^∗^Variables entered in the current discriminant analysis. Ϯ = *p* < 0.05, ns = nonsignificant (*p* > 0.05). EMS: external memory strategies, IMS: internal memory strategies, ACS: attentional control strategies, DS: distraction strategies, and RS: reappraisal strategies.

**Table 3:** Summary of interpretive measures for stepwise discriminant analysis.

Predictor	Standardized coefficient loadings	*F* ratio	Rank
Age	−0.626	15.239^∗∗^	1
Gender	0.413	1.469	
BMI	−0.307	3.824	
Perceived health status	0.442	4.230^∗^	4
Depression	−0.434	0.739	
Internal memory	0.213	10.684^∗^	2
Attentional control	−0.196	9.761^∗^	3
Barriers' self-efficacy	0.555	3.948^∗^	5
Canonical correlation	0.513		
Eigenvalue	0.358		
Wilks' lambda	0.736		
*χ* ^2^	72.051; df = 8		

^∗^ *p* < 0.01, ^∗∗^*p* < 0.001.

**Table 4:** Classification results of the discriminant analysis.

Group	Number of cases	Predicted group inactive *n* (%)	Membership active, *n* (%)
Inactive	104	63 (60.6%)	41 (39.4%)
Active	139	24 (17.2%)	113 (81.3%)

73.03% of grouped cases were correctly classified and 26.97% of grouped cases were incorrectly classified.
